# Tuberculosis caused by *Mycobacterium africanum*: Knowns and unknowns

**DOI:** 10.1371/journal.ppat.1010490

**Published:** 2022-05-26

**Authors:** Marta L. Silva, Baltazar Cá, Nuno S. Osório, Pedro N. S. Rodrigues, Ana Raquel Maceiras, Margarida Saraiva

**Affiliations:** 1 i3S - Instituto de Investigação e Inovação em Saúde, University of Porto, Porto, Portugal; 2 IBMC - Instituto de Biologia Molecular e Celular, University of Porto, Porto, Portugal; 3 Doctoral Program in Molecular and Cell Biology, ICBAS - Instituto de Ciências Biomédicas Abel Salazar, University of Porto, Porto, Portugal; 4 INASA - Instituto Nacional de Saúde Pública da Guiné-Bissau, Bissau, Guinea-Bissau; 5 Bandim Health Project, Indepth Network, Bissau, Guinea-Bissau; 6 Life and Health Sciences Research Institute (ICVS), School of Medicine, University of Minho, Campus Gualtar, Braga, Portugal; 7 ICVS/3B’s - PT Government Associate Laboratory, Braga/Guimarães, Portugal; University of Queensland, AUSTRALIA

## Abstract

Tuberculosis (TB), one of the deadliest threats to human health, is mainly caused by 2 highly related and human-adapted bacteria broadly known as *Mycobacterium tuberculosis* and *Mycobacterium africanum*. Whereas *M*. *tuberculosis* is widely spread, *M*. *africanum* is restricted to West Africa, where it remains a significant cause of tuberculosis. Although several differences have been identified between these 2 pathogens, *M*. *africanum* remains a lot less studied than *M*. *tuberculosis*. Here, we discuss the genetic, phenotypic, and clinical similarities and differences between strains of *M*. *tuberculosis* and *M*. *africanum*. We also discuss our current knowledge on the immune response to *M*. *africanum* and how it possibly articulates with distinct disease progression and with the geographical restriction attributed to this pathogen. Understanding the functional impact of the diversity existing in TB-causing bacteria, as well as incorporating this diversity in TB research, will contribute to the development of better, more specific approaches to tackle TB.

## Tuberculosis and tuberculosis-causing bacteria

Tuberculosis (TB) is one of the oldest, deadliest, and more devastating infectious diseases affecting humankind [1]. Despite the efforts made through the years and the progresses in diagnosis, treatment, and prevention of TB, this disease remains a public health threat. In 2020, around 10 million people fell ill with TB and an estimated 1.5 million died of TB [2]. For the first time in several years, and as a consequence of the Coronavirus Disease 2019 (COVID-19) pandemic, the notification of new TB cases decreased and the number of TB deaths increased [2]. Thus, an aggravation of the TB burden is expected in the next years [3]. Further hampering the goal of TB elimination are the enormous reservoir of latent TB-infected individuals, coinfections with the human immunodeficiency virus (HIV), and the emergence of drug-resistant strains [4,5]. Although TB affects all continents, over two-thirds of the reported cases are concentrated in Africa and Asia.

**Fig 1:**
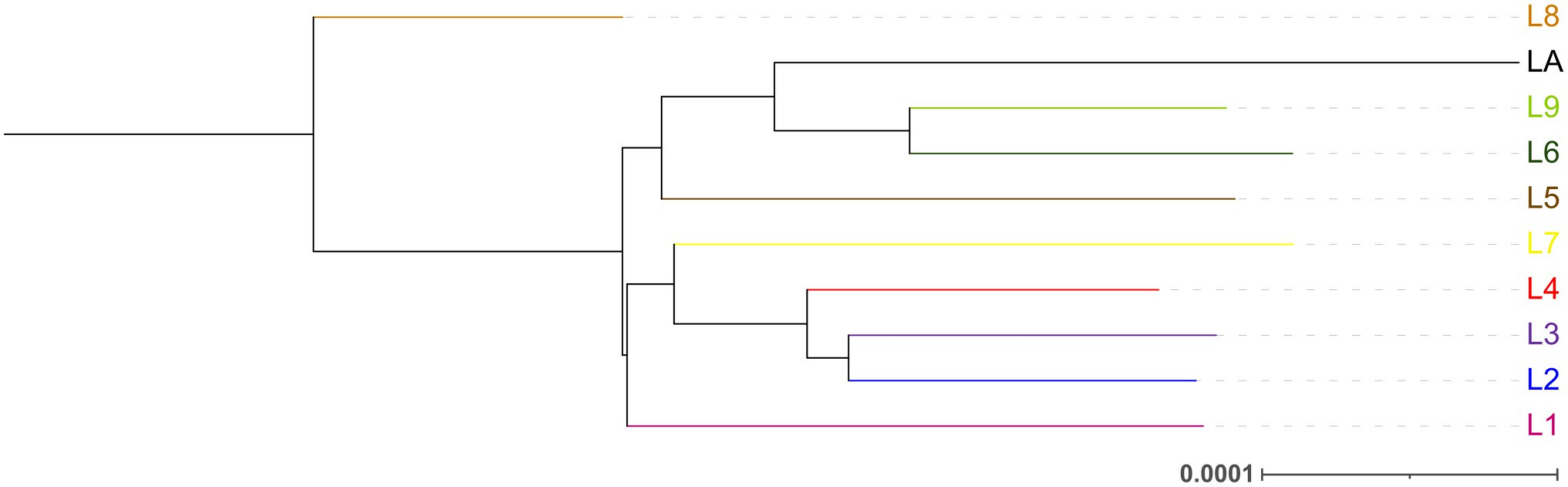
The phylogenetic structure of the MTBC. Phylogenetic analysis of 10 MTBC genomes selected to include 1 genome from each of the known MTBC lineages (accession numbers: SRR1162469, ERR2704812, ERR181314, SRR10828835, ERR1193734, SRR8237291, ERR3470572, ERR3470655, ERR756344, and ERR015582). A maximum likelihood tree was created with IQ-TREE v2.1.2 using TVM+F+I (the best-fit model of substitution according to AIC). The tree was coloured using iTOL 6.5.2 with the commonly used colour scheme for the different MTBC lineages: Lineage 1 (L1) in pink; Lineage 2 (L2) in blue; Lineage 3 (L3) in purple; Lineage 4 (L4) in red; Lineage 5 (L5) in brown; Lineage 6 (L6) in green; Lineage 7 (L7) in yellow; Lineage 8 (L8) in light brown; Lineage 9 (L9) in light green; and animal-associated lineages (LA) in black. Strains from L1–L4 and L8 are considered *M*. *tuberculosis sensu stricto* and L5, L6, and L9 *M*. *africanum*. The scale bar indicates the number of nucleotide substitutions per site. AIC, Akaike information criterion; MTBC, *Mycobacterium tuberculosis* complex.

Since Robert Koch’s initial identification of TB as an infectious disease, and of *Mycobacterium tuberculosis* as its causative agent, our understanding of the pathogen has greatly evolved. *M*. *tuberculosis* belongs to the *Mycobacterium tuberculosis* complex (MTBC), which comprises phylogenetically related TB-causing bacteria that differ in their host specificity [6]. Nine human-adapted lineages (L) belong to the MTBC: L1, 2, 3, 4, 7, and 8 are composed of strains classified as *M*. *tuberculosis sensu stricto*; strains belonging to L5, 6, and to the newly discovered L9 are classified as *Mycobacterium africanum* also known as *M*. *tuberculosis* var *africanum* [7–9]. Several sublineages have also been identified [9,10]. Strains belonging to L1 to L9 and the respective sublineages are considered human adapted because they are obligate pathogens of humans, without any known environmental or animal reservoir. However, they can still infect nonhuman hosts. Besides the 9 human-adapted lineages, the MTBC also includes bacteria that are highly adapted to cattle or several wildlife animals [11]. Among the animal-adapted members of the MTBC are *Mycobacterium bovis*, *Mycobacterium caprae*, and *Mycobacterium suricatae*. The phylogenetic structure of the MTBC is shown in Fig 1.

**Fig 2:**
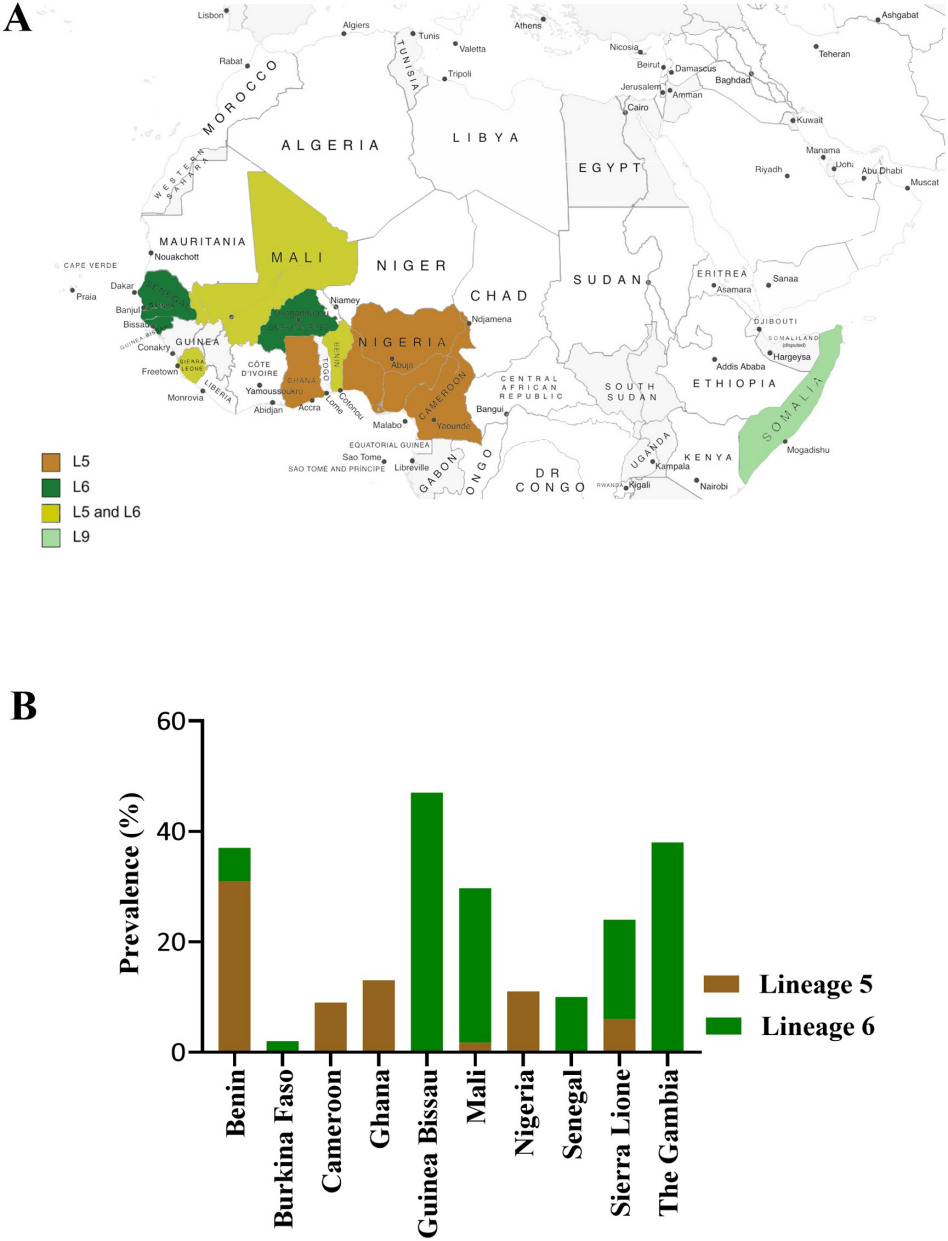
Geographic restriction and prevalence of *M*. *africanum*. (A) Geographic distribution of *M*. *africanum* lineages 5, 6, and 9 across Africa. Created with https://mapswire.com/africa/political-maps/. (B) Prevalence of lineages 5 and 6, according to the most recent studies conducted in each represented country [15–19].

Genetic analysis of the human-adapted TB-causing bacteria is suggestive of coevolution with the human host [6]. The evolution and out-of-Africa migration of early human populations are thought to have had an important role in the spread and establishment of strains of different *M*. *tuberculosis* lineages across the globe [12,13]. MTBC lineages and sublineages can be classified as generalists, if their distribution is widespread across the globe, or as specialists, if presenting a narrow geographic niche [10]. *M*. *tuberculosis* lineages are mostly classified as generalists with the exception of L7 that is restricted to Ethiopia in the horn of Africa [14], and L8 to the African Great Lakes region [8]. L4 is the most widely spread *M*. *tuberculosis* lineage, being the most prevalent in Europe and America, but also commonly found in all other continents [10,12]. Although L4 is mostly generalist, some L4 sublineages are considered specialists due to their restricted geographic distribution [10]. In contrast, all known *M*. *africanum* lineages (L5, 6, and 9) and sublineages are geographically restricted to specific regions of Africa (Fig 2A). L5 strains appear mostly in the east side of West Africa, with a high prevalence in countries as Benin and Ghana [15,16], while L6 strains are more prevalent in the west part of West Africa, affecting countries as Guinea Bissau [17], Sierra Leone [18], and The Gambia [19]. L9 strains have so far only been isolated in Somalia [9]. *M*. *africanum* is an important cause of TB in West Africa, where it is estimated to cause up to half of all TB cases [20] (Fig 2B). A decline of the incidence of *M*. *africanum* has been reported over time for some countries [21–23], although not for others [17,23]. It will be interesting to monitor the dynamics of *M*. *africanum* by continuous assessment of its prevalence in West Africa. A recent study has reinforced the importance of *M*. *africanum* strains as a cause of TB in the Volta region of Ghana, particularly in less cosmopolitan areas [24]. TB caused by *M*. *africanum* strains outside West Africa, although very rare, has been described in several countries [25–28]. Importantly, these cases were mostly found in migrants from endemic areas in Africa, reinforcing the idea that *M*. *africanum* may be restricted to certain host ancestries [29]. However, exceptions to this have been reported. In a study in California, 2 of 5 patients diagnosed with *M*. *africanum* did not present epidemiologic relation to *M*. *africanum* endemic areas [30]. Similarly, in Canada, of the 4 *M*. *africanum* TB cases reported between 2004 to 2015 [31], 2 of them were of Canadian-born patients with no travel history to West Africa [31]. Additionally, in 2020, a case of *M*. *africanum* in a Brazilian woman without association with Africa was reported in Brazil [32]. Although not all these reports provided whole-genome sequence analysis to assess the existence of transmission clusters, they do evoke the possibility of local transmission of *M*. *africanum* outside endemic areas. It would be very interesting to dissect the host genetic and immune characteristics across these different cases, in search for potential susceptibility factors associated with *M*. *africanum* infections.

## The identity of *M*. *africanum*

**Fig 3:**
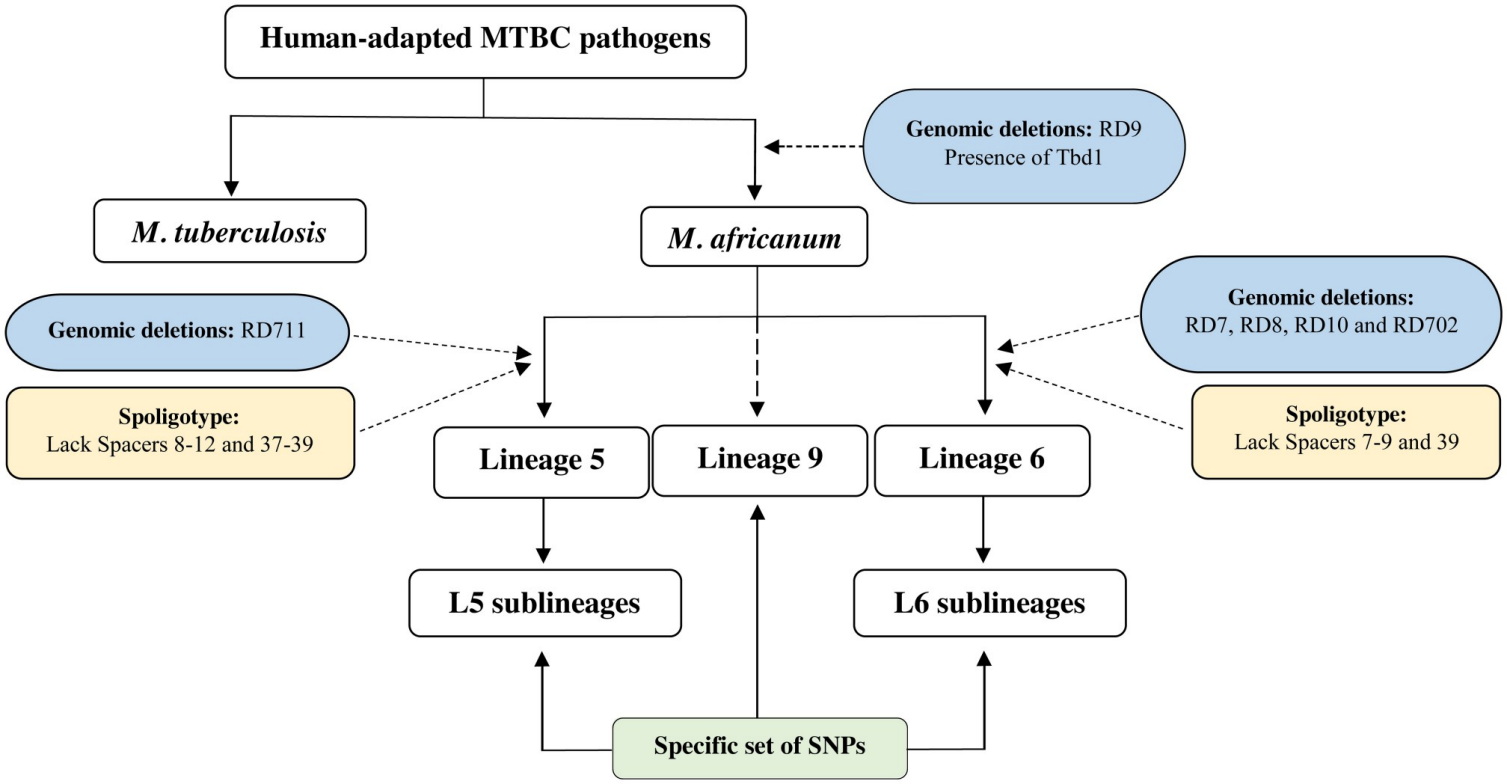
Genetic identity of *M*. *africanum* lineages. Represented are the genetic markers that distinguish *M*. *africanum* from *M*. *tuberculosis* and lineages 5, 6, and 9 within *M*. *africanum*. Of note, since no robust genetic deletions allowing the classification of L9 have been reported, the identification of this lineage is based in specific SNPs. L, lineage; MTBC, *Mycobacterium tuberculosis* complex; RD, region of difference; SNP, single nucleotide polymorphism.

*M*. *africanum* strains were initially divided into 2 main subtypes based on their geographic origin: *M*. *africanum* subtype I, for strains originating from West Africa and *M*. *africanum* subtype II for strains from East Africa [33]. Subsequent genetic studies placed strains of *M africanum* subtype II, also known as the “Uganda genotype,” as belonging to a sublineage of L4, thus leaving *M*. *africanum* strains restricted to West Africa [33,34]. More recently, comparative genomics approaches established the presence or absence of specific genomic regions from the common ancestor in *M*. *tuberculosis* and *M*. *africanum*, allowing *M*. *africanum* strains to be subdivided into 2 genetically distinct lineages: L5 and L6 (previously known as Maf1 and Maf2, respectively) [12,35] (Fig 3). *M*. *tuberculosis* and *M*. *africanum* strains are readily distinguishable by the deletion of the region of difference (RD) 9 in the genome of *M*. *africanum* strains. The genome of strains within L5 has a further deletion in RD711, while genomes of strains within L6 are characterized by a deletion of RD702 [35]. Through spoligotyping, L5 and L6 can be respectively identified by the loss of spacers 8 to 12 and 37 to 39 (L5), and 7 to 9 and 39 (L6) in their genomes [19,35]. Together, these genomic deletions can be used as phylogenetic markers distinguishing *M*. *africanum* from *M*. *tuberculosis* strains and allowing the genomic identification of L5 and L6 strains (Fig 3). More recently, with the increasing availability of multilocus and whole-genome sequencing and analyses, several specific SNPs in the genome of strains belonging to L5 and L6 have been identified, which are the basis of SNP assays for rapid identification of strains belonging to L5/L6 *M*. *africanum* lineages [36,37]. Whole-genome analyses also revealed the existence of genetic diversity within *M*. *africanum* L5 and L6 strains [9,38–40] and allowed the construction of detailed MTBC phylogeny trees. It is interesting to note that strains of the *M*. *africanum* L6 are separated from the other lineages of human-adapted members of the MTBC and instead placed among the animal lineages of the MTBC (Fig 1) [41]. Furthermore, several studies support the idea that the dassie bacillus shared a common ancestor with *M*. *africanum* L6 [41,42]. In all, data are compatible with an evolutionary scenario in which the ancestor of L6 strains was a generalist pathogen that subsequently adapted to different host species, and with the hypothesis that L6 strains may originate from an animal reservoir, as further discussed below.

The most recently proposed *M*. *africanum* lineage, L9, resulted from the analysis of 675 *M*. *africanum* genomes, of which 5 could not be classified into any of the known MTBC lineages [9]. L9 appears as a sister clade of L6, being placed between L6 and the animal-adapted lineages. Genomes of strains belonging to L9 share some genomic deletions with those of strains belonging to L6, as RD702, but not others also present in genomes of strains of animal-associated lineages, such as RD1 and RD5. Owing to its distinct geographic location and the still significative genetic separation from L5/L6 strains genomes, a new *M*. *africanum* lineage was thus proposed [9]. Of note, phylogenetic markers to identify L9 based on genomic deletions have yet not been described (Fig 3). Instead, a list of SNPs common to all L9 genomes and absent from other lineages was proposed as phylogenetic markers for L9 [9]. This can now be used as a framework for the discovery of other strains within L9, as well as for further functional studies of this lineage.

Given the close phylogenomic proximity of *M*. *tuberculosis* and *M*. *africanum* strains these 2 pathogens may be considered as belonging to the same species [43,44], which is still a matter of debate. Of note, the phylogeographic distribution and the host preference of the different members of the MTBC suggest that this complex could represent host-adapted ecotypes [45]. Also, there are important molecular and phenotypical differences between *M*. *tuberculosis sensu stricto* and *M*. *africanum* (Table 1). Initial studies showed that unlike *M*. *tuberculosis*, *M*. *africanum* strains are unable to use glycerol as a sole carbon source, a characteristic similar to *M*. *bovis*, which implies the need to supplement the culture medium with pyruvate in laboratory cultures [46]. A recent study confirmed the preference of *M*. *africanum* L5 and L6 strains for pyruvate over glycerol as a carbon source, even though L5 also grows in media with glycerol [47]. Furthermore, several studies show that, as compared to *M*. *tuberculosis*, *M*. *africanum* strains present a slower growth rate in axenic medium, which is particularly visible in the case of L6 [48–51]. A lower growth rate for both L5 and L6 strains as compared to L4 ones was also documented in 7H11 solid medium and in 7H9 liquid medium [47]. Also remarkable is the evidence that *M*. *africanum* strains prefer minimal oxygen microenvironments (microaerophilic) and produce dysgonic colonies, contrary to *M*. *tuberculosis* strains that exhibit eugonic colonies [52]. Moreover, *M*. *africanum* L6 strains are adapted to growth under hypoxic conditions [53]. Metabolic differences have also been described between *M*. *tuberculosis* and *M*. *africanum* strains. The nitrate reductase activity is strong in the case of *M*. *tuberculosis* strains and weak to negative for both L5 and L6 strains [54–56], and *M*. *tuberculosis* L4 and *M*. *africanum* L6 strains present a stronger average urease activity, which is directly linked to the bacteria nitrogen metabolism, than L5 [47]. Therefore, globally, L5 and L6 strains present several characteristics that are distinctive of *M*. *tuberculosis*. No phenotypic data pertaining L9 strains exist thus far, and so it is as yet not possible to include L9 strains in these comparisons. Hence, the classification of TB-causing isolates into the different lineages of *M*. *tuberculosis* or *M*. *africanum* based in genotypic differences (Fig 3) is more accurate than one based on phenotypic assays, also because one cannot exclude phenotypic diversity among isolates of the same lineage or even sublineage.

**Table 1:** Main differences discriminating *M*. *africanum* and *M*. *tuberculosis*.

	*M*. africanum* L5	*M*. *africanum* L6	*M*. *tuberculosis*	Ref.
**Growth characteristics**				
Carbon source	Glycerol/pyruvate	Pyruvate	Glycerol	[46,47]
Growth rate	Slow	Slowest	Fastest	[47–51]
Depth of growth	Microaerophilic	Microaerophilic	Aerophilic	[52,53]
Colony morphology	Dysgonic	Dysgonic	Eugonic	[52]
**Biochemical characteristics**				
Nitrate reductase	Negative/weak	Negative/weak	Positive	[54–56]
Urease activity	Positive (high)	Positive	Positive	[47]
**Geographical distribution**	Mainly east side of West Africa	Mainly west side of West Africa	Widespread	[10,15–19]
**Clinical characteristics**				
Sputum conversion		Slower**		[61]
Reported associations		Elder, HIV+, and malnutrition		[60,63,64]
Rates of transmission	Reduced**	Similar**		[66,69]
Progression to active TB		Slower**		[64,66]

*M. *africanum* L9 is not included due to lack of data.

**As compared to *M*. *tuberculosis*.

At the genetic level, *M*. *africanum* strains present important variations in some “classical” virulence factors of *M*. *tuberculosis*. An example are the DosR regulon genes, which have been linked to virulence in the MTBC strains, and shown to be down-regulated in the sputum of *M*. *africanum* L6-infected patients as compared to infections by *M*. *tuberculosis* L4 [53]. *M*. *africanum* strains accumulate mutations in major DosR regulon genes, and the lower expression of the DosR regulon may explain why *M*. *africanum* strains present microaerobic growth and associates with extrapulmonary disease [53]. Strains of the *M*. *africanum* lineages were also described to accumulate mutations in genes encoding enzymes of the electron transport chain and central carbon metabolic pathways, as compared to *M*. *tuberculosis* strains, which has been suggested as another possible adaptation to ecological niches characterized by low oxygen tension [57]. Furthermore, *M*. *africanum* L6 strains were shown to harbor loss-of-function mutations in the 2-component virulence regulation system phoP/R [58,59]. Because this system is involved in several pathogenic processes, as the secretion of the virulence factor ESAT-6, biosynthesis of acyltrehalose-based lipids, and the modulation of antigen export, such mutations were expected to render the pathogen avirulent. However, *M*. *africanum* L6 strains evolved to compensate the deleterious effects of the *phoP/R* mutations and so maintain their pathogenic ability. Indeed, the RD8-specific deletion in both animal-adapted and *M*. *africanum* L6 strains restores ESAT-6 secretion by a phoP/R-independent mechanism, by ensuring high levels of expression of the operon *espACD*, which is required for ESAT-6 secretion [58,59]. How this occurs is not fully understood, but it is possible that polymorphisms upstream the *espA* gene might increase the affinity of PhoP or EspR for this promoter region, which results in *espA* expression in the absence of a fully functional phoP/R system [58]. In a different study, *M*. *africanum* L5 isolates were also shown to efficiently secrete and induce immune responses against ESX-1 substrates [38].

## TB caused by *M*. *africanum* versus *M*. *tuberculosis*

Several studies have been performed with the aim of unveiling associations between clinical and epidemiologic data and the infecting bacteria, i.e., *M*. *tuberculosis* or *M*. *africanum*. It is important to note that these studies have been performed in different countries, at different times, and using different methodologies. Therefore, multicentric studies are still in need, and it is somehow not surprising that some discrepant results are seen across different reports, as further discussed below.

No marked differences were found in the chest X-ray presentation of TB caused by *M*. *africanum* or *M*. *tuberculosis* [60]. Furthermore, both pathogens were shown to respond similarly to the standard 4 first-line drugs in TB treatment, although patients diseased with *M*. *tuberculosis* L4 strains responded faster to TB treatment than those with *M*. *africanum* L6 strains [61]. The slow clinical recovery of *M*. *africanum*-infected patients as compared to *M*. *tuberculosis*-infected ones may result from a higher content of persister-like *M*. *africanum* bacilli in sputum at diagnosis [62]. Despite the similar clinical presentation of TB, several studies associated infections with *M*. *africanum* strains with more vulnerable hosts. Studies conducted in The Gambia found *M*. *africanum* infections to be more common in HIV-coinfected patients, as well as in older individuals and individuals presenting severe malnutrition [60]. The association between *M*. *africanum* infection and elder patients was also reported in Ghana [63] and with lower body mass index individuals in Mali [64]. However, a clear association between *M*. *africanum* and HIV coinfection is still controversial. A recent study in Ghana showed no significant differences between the prevalence of *M*. *tuberculosis* or *M*. *africanum* infections in individuals with diabetes, another important comorbidity in TB [65]. Furthermore, it is possible that *M*. *africanum* infections correlate with slower progression to active TB. A study from The Gambia showed that despite similar rates of transmission, individuals exposed to *M*. *tuberculosis* strains were more likely to progress to active TB disease than those infected with *M*. *africanum* ones [66]. This was supported by another study in Mali associating a longer time between symptom onset and TB diagnosis in *M*. *africanum* infections [64]. In line with these findings, infection of mouse models with *M*. *africanum* strains showed a slower progression of the disease [51,67,68] with mild lung pathology even in mice lacking IFN-γ, which are highly susceptible to *M*. *tuberculosis* infection [51]. Of note, whether transmission rates are equivalent between *M*. *tuberculosis* and *M*. *africanum* is not fully set, as a study in Ghana associated *M*. *africanum* strains with reduced recent transmission rates [69]. Importantly, whereas in The Gambia, the prevalent *M*. *africanum* lineage is L6 [66], in Ghana, it is L5 [69], and so differences in transmission rates may reflect the specific characteristics of L5 or L6 strains. Thus, all in all, *M*. *africanum* strains present several differences when compared to *M*. *tuberculosis* ones (Table 1) and is generally viewed as a less virulent pathogen than *M*. *tuberculosis*. As mentioned before, it is as yet not possible to establish comparisons between the recently identified L9 strains and those of L5/L6 or *M*. *tuberculosis*.

## Host immune responses to *M*. *africanum*

### Innate immune responses

Infection of human monocyte-derived macrophages with distinct strains of the MTBC showed variation of the induced cytokine responses including between the 2 isolates of *M*. *africanum* tested, with 1 inducing strong cytokine release and another inducing a weak response [70]. Interestingly, in the same study, both *M*. *africanum* isolates seemed to grow less inside resting macrophages than their *M*. *tuberculosis* counterparts [70]. Another study, focusing on the pathogen transcriptional adaptation upon macrophage infection, reported distinct MTBC lineage signatures, including the failure of *M*. *africanum* strains to induce the phthiocerol dimycocerosate (PDIM) locus, a complex cell wall lipid unique to mycobacteria associated with its virulence [71]. More recently, an isolate of *M*. *africanum* L6 was shown to induce considerably less IFN-β by infected bone marrow–derived macrophages than *M*. *tuberculosis* strains from L2 or L4 [72]. Although the *M*. *africanum* isolate also triggered cGAS and STING, infections of macrophages by this pathogen induced less mitochondrial stress, thus decreased production of mitochondrial reactive oxygen species that contributed to less type I IFN being produced [72]. The in vivo effect of IFN-αβ signalling during infection by *M*. *africanum* strains was subsequently studied in mouse models. In agreement with the detrimental role of type I IFN in TB [73], lack of type I IFN receptor signaling led to reduced lung bacterial burdens and less severe histopathological findings upon *M*. *africanum* infection [67]. These results highlight that even the lowest levels of IFN-αβ induced during chronic *M*. *africanum* infection are potentially pathogenic [67]. Collectively, these studies and others [51] demonstrate that *M*. *africanum* strains infect macrophages, inducing a cytokine response, while adapting to the host cell. The molecular mechanisms underlying these responses, such as the recognition of *M*. *africanum* strains by pattern recognition receptors, remain however elusive. *M*. *africanum* strains were shown to bind recombinant human mannose-binding lectin (MBL), a plasma opsonin, more efficiently than *M*. *tuberculosis* strains and a protective association between TB and the human MBL2 G57E variant, associated with lower MBL levels, was described, only in TB caused by *M*. *africanum* [74]. It is possible that the stronger binding of *M*. *africanum* strains to MBL may favour the bacteria uptake by macrophages, promoting the establishment of infection in vivo, and thus the protective MBL deficiency may have been selected in the human population in regions endemic for *M*. *africanum*. Another study has identified increased levels of *TLR9* expression in unstimulated blood of patients infected with *M*. *africanum* isolates as compared to other MTBC strains infections [75], which may suggest a role for TLR9 in innate immune responses to *M*. *africanum* strains. Of note, the levels of IL-12p70 and *IL12A* were also significantly higher in *M*. *africanum*-infected patients, while those of IL-15, IL8, and MIP-1α were higher in *M*. *tuberculosis*-infected patients [75]. A broader study comparing peripheral blood gene expression profiles between *M*. *africanum*- and *M*. *tuberculosis*-infected patients showed no differences at diagnosis, although there were distinct signatures associated with each infection posttreatment, predominantly associated with immune responses and metabolic diseases [76].

### Adaptive immune responses

Comparison of T cell responses from *M*. *tuberculosis*- or *M*. *africanum*-infected TB patients before chemotherapy and following overnight stimulation of whole blood with ESAT-6/CFP-10 or with purified protein derivative (PPD) showed higher single-TNF-α-producing CD4 and CD8 T cells and lower single-IL-2-producing T cells in the case of *M*. *africanum* infections [77]. Additionally, a persistently high proportion of activated T cells was reported in *M*. *africanum*-infected individuals posttreatment [77]. However, the frequencies of PPD-specific polyfunctional CD4 T cells did not differ between the 2 infections [77], both before and after treatment, suggesting an overall uniform immune response triggered by either pathogen. This is in line with the abovementioned studies on peripheral blood transcriptomic and metabolic profiles obtained at diagnosis [76]. Interestingly, stimulation of whole blood with ESAT-6/CFP-10 stimulation after treatment induced significantly higher production of pro-inflammatory markers, such as IFN-γ, in the case of *M*. *tuberculosis*-infected TB patients [75,76]. In the mouse model of infection, a modest immune response has been reported upon infection with a *M*. *africanum* isolate, also associated with restricted lung pathology [51]. Taken all this together, it is possible that a lower immune response takes place upon *M*. *africanum* infection, which although precluding the clearance of the pathogen, may protect the host from tissue immune pathology. This hypothesis is compatible with a slower progressing infection and may be explained by pathogen-associated factors. Pathogens belonging to the MTBC are known to have remarkably hyperconserved T cell epitopes, suggesting that ensuring T cell responses is more important to these agents than evading them [78]. Interestingly, a study showed that the L6 strains of *M*. *africanum* were significantly more genetically diverse than the L5 ones, including in predicted T cell epitopes [79]. Additionally, even though the majority of the T cell epitopes were conserved between the 2 lineages, a higher ratio of nonsynonymous to synonymous single nucleotide variation was detected in the epitopes from L6 strains relatively to L5 ones [39]. Thus, it is tempting to speculate that the evolutive pressure to hyperconserve T cell epitopes may be weaker in the case of L6 strains, leading to lower T cell responses and favouring the persistence of the pathogen in its host population. Further studies are required to address these hypotheses and investigate the contribution of T cell responses to TB caused by *M*. *africanum* strains.

## Geographic restriction of *M*. *africanum*: A case of immune adaptation?

A specific adaptation of *M*. *africanum* to the host population, particularly to the host immune response, is a conceivable hypothesis to explain the geographic restriction of this pathogen. Previous studies provide compelling evidence towards this hypothesis in the case of *M*. *africanum* L5 strains. In a study in Ghana, *M*. *africanum* was significantly more common in TB patients belonging to the Ewe ethnic group an association that was mainly driven by L5 strains [80]. Possible interactions between *M*. *africanum* infection and human genetic diversity were also described in other studies. A polymorphism in the exonic allele (g.760A) of the ALOX5 gene (which encodes for 5-lipoxygenase, an important regulator of the immune response in TB [81,82]) was associated with higher risk of TB in Ghana, an association that was stronger in infections caused by *M*. *africanum* L6 strains [83]. Furthermore, another study identified a highly frequent variant of the human immunity–related GTPase M (IGRM), a regulator of the autophagic process, in the Ghanaian population and associated it with protection against *M*. *tuberculosis* L4 strains, but not against *M*. *africanum* isolates [84]. Thus, higher frequencies of genetic variants conferring increased susceptibility to *M*. *africanum* strains in West African individuals may at least partially explain the geographical restriction of this pathogen to this region. Still, more studies linking human and pathogen genetic diversities are needed to validate this hypothesis. In this line, specific HLA genetic associations may be of potential interest to explain the geographic distribution of *M*. *africanum* versus *M*. *tuberculosis* infections. A study conducted in Mali identified various class I HLA-A and HLA-B alleles associated with active TB disease caused by either pathogen. However, several class II HLA-DR variants were found to be associated with *M*. *tuberculosis* but not *M*. *africanum* strains, with only the variant DRB1*03:01 being associated with both groups [85]. It is tempting to speculate that specific associations between HLA variants and *M*. *africanum* strains may reflect variations affecting T cell epitopes in *M*. *africanum*, which as described above are not as hyperconserved as in *M*. *tuberculosis*.

More recently, the hypothesis that differences in the intestinal microbiota of patients infected with *M*. *africanum* isolates could contribute to the high susceptibility of West African individuals to infections with this pathogen has been proposed [86]. Patients infected with *M*. *africanum* strains presented less microbiome diversity than individuals infected with *M*. *tuberculosis* isolates or healthy controls and were enriched in bacteria from the Enterobacteriaceae phylum Proteobacteria as compared to healthy controls [86]. Since a positive correlation between the abundance of Enterobacteriaceae and an inflammatory gene expression profile was reported, differences in the intestinal microbiome may contribute as host-associated factors predisposing to infections by *M*. *africanum*.

Other 2 hypotheses may explain the geographic restriction of *M*. *africanum* infections, which are less related to the host immune response. It is possible that *M*. *africanum* is an attenuated member of the human-adapted TB-causing bacteria, being therefore outcompeted by *M*. *tuberculosis*. This hypothesis is supported by the reduction of TB cases caused by *M*. *africanum* strains over time for some countries [21–23], although it is not observed in other countries [17,23] as discussed above. Finally, *M*. *africanum* L6 strains share a common ancestor with animal (nonhuman) adapted strains (Fig 1). Animal-adapted lineages are composed of Mycobacteria that infect different species of animals as preferential hosts, including nonhuman primates and other mammals. The ancestry of *M*. *africanum* L6 allows raising the hypothesis that, despite having become a human pathogen, strains of this lineage may still be adapted to an animal that could function as a reservoir in West Africa. Although *M*. *africanum* strains have been isolated from several animal species, including pigs and cows [87], an animal reservoir has never been identified. Of note, the animal reservoir hypothesis is less likely to prove valid in the case of L5 strains since this lineage is phylogenetically less related to animal adapted members of MTBC [41] (Fig 1).

## Conclusions

Up to 50% of the TB cases in West Africa have been attributed to *M*. *africanum* strains. A striking feature characterizing these TB-causing bacteria is its geographical restriction, which contrasts with the widespread distribution of *M*. *tuberculosis* strains and remains largely unexplained. Adaptation of *M*. *africanum* strains to the West African population, perhaps mediated through differential modulation of the immune response, is a likely hypothesis. Importantly, infections of humans and experimental models with strains of *M*. *africanum* are generally more attenuated than those with *M*. *tuberculosis* strains. This offers an opportunity to learn from *M*. *africanum* and its interactions with the host, with the aim of better controlling *M*. *tuberculosis*. There are several outstanding questions that would advance our knowledge in this field towards better strategies to tackle TB:

1. What is the actual origin of *M*. *africanum*? Are there nonhuman reservoirs relevant in supporting human transmission? This would provide important information on the evolution of specific members of the MTBC, as well as potentially guide measures to mitigate *M*. *africanum* infections.
2. Are *M*. *tuberculosis* and *M*. *africanum* distinct entities? This remains a matter of debate, as although recent studies suggest that both pathogens belong to the same species, phenotypic differences between *M*. *tuberculosis* and *M*. *africanum* strains are well documented. Additional studies are required to fill this knowledge gap further informing similarities and particularities of different MTBC members of relevance for TB management.
3. Are there coinfections caused by *M*. *africanum* and *M*. *tuberculosis* strains? The fact that both pathogens are endemic in the same geographic area would suggest a scenario where coinfections are possible. Clarifying this question would be interesting to understand which pathogen would impact more disease presentation or if different clinical/immune response characteristics would prevail.
4. What are the differences between TB and the immune response during infection with L5, L6, and L9 strains? How does this correlate with *M*. *tuberculosis* infections? Does *M*. *africanum* modulate innate or T cell responses in specific populations? Is this associated with increased susceptibility of the population or decreased virulence of the pathogen? Disclosing the immune signatures of *M*. *africanum* infections and correlating those with the disease manifestation will provide valuable knowledge to develop potential immune interventions in TB, including vaccines.
5. Are there differences in latency establishment, duration, or reactivation between *M*. *tuberculosis* and *M*. *africanum* strains? Elucidating this question is hampered by our inability to categorize the pathogen species in latent cases. However, by revealing immune signatures of *M*. *africanum* infections, it may be possible to then look at latent and progressing cohorts in an attempt to establish latent signatures specific of the different MTBC members. This will further our knowledge on the natural history of TB, again potentially offering novel targets to control TB.

Answering these questions to understand the differences between *M*. *africanum* and *M*. *tuberculosis* strains will provide valuable knowledge towards identifying the cellular and molecular determinants allowing the widespread of the *M*. *tuberculosis* strain lineages, which are a global threat. This knowledge will also advance our understanding on the biology of *M*. *africanum* and its interactions with the human host, which is highly relevant considering the TB burden in West Africa. Furthermore, consistently stratifying for the type of infecting bacteria in human-based studies will contribute to a better interpretation of novel TB intervention tools, including diagnosis and vaccines. All this will in turn inform the development of better, more specific approaches to tackle TB.
